# The potential of early years’ childcare to reduce mental health inequalities of school age children in Scotland

**DOI:** 10.1016/j.ssmph.2024.101682

**Published:** 2024-05-23

**Authors:** Elaine Robertson, Alastair Leyland, Anna Pearce

**Affiliations:** MRC/CSO Social and Public Health Sciences Unit, School of Health and Wellbeing, University of Glasgow, Clarice Pears, Building, 90 Byres Road, Glasgow, G12 8TB, UK

**Keywords:** Health inequalities, Mental health, Childcare, Early years, Life course

## Abstract

Preschool childcare is considered an important policy for reducing inequalities in children's cognitive and socio-emotional development, although the population-level benefits for children under three years, is less clear. We examined the potential for childcare across the whole early years' period to benefit mental health and reduce inequalities, under different hypothetical policy scenarios, in the Growing Up in Scotland study.

Marginal structural logistic regression models estimated odds ratios (ORs) to quantify inequalities in mental health and consider how these would be altered under different hypothetical scenarios. Mental health (the outcome) was measured using the total Strengths and Difficulties Questionnaire score at the start of primary school. Socioeconomic circumstances (the exposure) were represented by maternal educational measured in infancy. Sequence analysis identified common patterns of childcare usage from 10 months to four years (the mediator). Confounders were adjusted for using inverse probability of treatment weights and analyses accounted for sampling design and attrition (complete case sample, *n* = 3205).

With virtually universal uptake of government-funded childcare at 3–4 years, most variation was seen before age three. Four groups were identified: ‘Parents, family & friends’ (35.8%), ‘Grandparents’ (32.7%), ‘Private group childcare’ (e.g. nurseries 23.5%), ‘Single professional care’ (e.g. childminders 8.1%). Children whose mothers had low, compared to high, educational qualifications were 3.18 times more likely to have mental health problems (95% CI: 1.88–5.37). In a hypothetical scenario where everyone received private group childcare, inequalities increased slightly to 3.78 (95%CI: 1.46–9.76). In an alternative scenario, where everyone received single professional childcare, inequalities in mental health reduced to 2.42 (95% CI: 0.20–28.76), albeit with wide confidence intervals.

Universal childcare provision before three years may widen or narrow socioeconomic inequalities in children's mental health, depending on the childcare type provided. Further research is required to understand the role of childcare quality, which we were unable to account for.

## Highlights

• Sequence analysis was used to understand childcare usage in early years.
• Childcare provision for under 3's is more heterogeneous than for over 3's.
• Causal mediation analysis predicted hypothetical policy impacts on child outcomes.
• Non-parental care under 3 could widen or narrow child mental health inequalities.
• Depending on the type of childcare used, potentially due to variations in quality.

## 1 Introduction

The early years is a particularly important time in a child's life for physical growth, cognitive development and socioemotional learning (Pearce et al., 2020). Universal childcare provision aims to ensure that all children, regardless of their family circumstances, start school ready to engage and learn through having the requisite developmental, socioemotional, and cognitive skills (Hall & Stephens, 2020; Melhuish et al., 2015; Scottish Government, 2022b). In addition, universal childcare is a means of enabling parents, particularly mothers, to return to education, training, and employment (Melhuish, 2004; Scottish Government, 2019, 2022b). This in turn seeks to improve family socioeconomic circumstances in the longer term. In acknowledgement of this wide-ranging benefits, many high-income countries have committed to providing high quality early years learning and childcare (Gromada & Richardson, 2021).

Drawing on framework around the mechanisms through which health inequalities can arise (Diderichsen et al., 2019), childcare has the potential to impact upon inequalities in children's outcomes via *differential exposure* (Diderichsen et al., 2001), or in other words, if there are inequalities in the uptake of childcare. Many governments have striven to provide universal childcare, at no or subsidised cost, to families with young children. For example, in Scotland, some universal childcare is in place for all three to four-year old children and 97% of children take this up (Knudsen et al., 2017). Free places are also made available to two-year olds from disadvantaged backgrounds (mygov.scot, 2022) approximately 14% of all two-year olds are registered for funded childcare in Scotland, it is estimated that around 25% of two-year olds are eligible (Scottish Government, 2022c). Thus, these policies are intended to reduce differential exposure in a way that could benefit inequalities. However, the types of childcare families use is, in practice, also affected by local and family-level factors, including the availability of places, age ranges covered by providers, opening hours, geographic location, and cost (which is relevant to wrap-around care, since parents often require longer hours than those offered through universal entitlements) (Chen & Bradbury, 2020). These practical considerations are not static and depend upon the changing needs and resources of a family over time, meaning that childcare use can be dynamic.

A second mechanism through which childcare might widen or reduce inequalities in outcomes is *differential susceptibility*, which refers to when the benefits (or detrimental impacts) of childcare on outcomes vary by SECs. (Diderichsen et al., 2001; Pearce et al., 2019). These differential benefits may occur if there are differences in childcare quality. There is strong evidence of overall benefits of high quality formal childcare to children's socioemotional, cognitive, language and educational development for children between the ages of three to five years (D'Onise et al., 2010; Melhuish et al., 2015). Formal childcare is also associated with benefits for those from the most disadvantaged communities (Barnett, 2008; McLaughlin et al., 2007; Melhuish et al., 2015; Schweinhart et al., 1985). Knowledge around the impacts of childcare provision and childcare quality on children's outcomes before the age of three is more heterogenous. Early research suggested that early childcare, particularly group childcare, could negatively affect children's socioemotional development, potentially due to negative impacts on maternal attachment (Belsky & Rovine, 1988; Clarke-Stewart, 1989). More recent studies have found that *high quality* childcare attendance (compared to parent only care) before the age of two years had a positive impact on language, although with no evidence of impact on motor skills and a negative impact on behaviour. Importantly, the positive impact on language skills is stronger for children from the most deprived backgrounds compared to those from least deprived (Berger et al., 2021). In a review, Melhuish et al. (2015) found that early formal childcare can be positive for children facing higher levels of deprivation, particularly in cognitive and language development. However few studies have formally compared the benefits of different types of childcare before the age of two years across different socio-economic groups, with the majority focussing only on socially deprived samples. One exception is a study using the UK Millennium Cohort Study. Researchers found that access to centre-based childcare between 26 and 32 months of age decreased the risk of poor socioemotional health for children in the lowest socioeconomic circumstances more than for children in the highest socioeconomic circumstances, therefore potentially attenuating inequalities (Green et al., 2021). This study estimated the impacts of centre-based and non-centre-based childcare across the ages of 26–31 months. The current study takes a longitudinal perspective accounting for childcare usage throughout the entire early years period. We examine the most common patterns of childcare use which were used by families throughout this period and thus additionally differentiate between care by single professionals and grandparents.

The Scottish Government (2021) are seeking to expand the funding of universal childcare to one and two year old children in low income households, with the 10.13039/100013986UK Government also recently pledging to extend the universal entitlement to all working parents of under twos (Department of Education, 2023). The main aims of this research were to create a longitudinal picture of childcare in the early years and estimate the hypothetical impact of different childcare scenarios on inequalities in children's mental health in Scotland. We focus on mental health as inequalities are evident from an early age (Piotrowska et al., 2015; Reiss, 2013) with potential to widen inequalities, in life chances (Campion et al., 2013).

## 2 Methods

### 2.1 Data

Growing Up in Scotland (GUS) is a longitudinal study that follows the children and their families since 2005 (ScotCen Social Research, 2019a). GUS data was shared under Special Licence Access via UK Data Service (ScotCen Social Research, 2019b). We use data from GUS ‘birth cohort 1’, which follows 5217 children from the age 10 months (ScotCen Social Research, 2019a). Face-to-face CAPI (Computer Assisted Personal Interview) interviews were held with the cohort child's main carer at every sweep and with cohort children from the age of 8 (ScotCen Social Research, 2019a). Response rates for GUS have declined over time to n = 3657 (70.1% of baseline) by the time cohort children started primary school in sweep 6 (the last sweep used in the current analysis). The initial baseline data collection was subject to medical ethical review by the Scotland ‘A’ MREC committee (application reference: 04/M RE 1 0/59) and via substantial amendment submitted to the same committee for subsequent sweeps.

### 2.2 Exposure, mediator and outcome measures

Highest level of maternal education was categorised using the Scottish Credit & Qualifications Framework to represent socio-economic circumstances as the main exposure variable (degree/Highers/Upper-level Standard grades/lower-level Standard grades or none). Within GUS, 95% of the respondents were the child's mother resulting in high levels of completion. At the time, in Scotland, Standard grades were taken around the age of 16 years and were designed to be a national qualification for those who did not want to pursue further academic study after the age of 16 years. Highers were the highest level of school qualification taken at 18 years and were broadly equivalent to A-levels in England or high school diplomas internationally. Maternal education is considered a reliable measure of SEC (Galobardes et al., 2006), and has low level of missingness compared to other measures such as household income. A sensitivity analysis was also conducted using National Statistics Socioeconomic Classification (NS-SEC), measuring highest occupation in the GUS child's household and this produced similar results to maternal education.

Mental health, the outcome, was measured using the Total Difficulties (TD) score of Goodman's Strengths and Difficulties Questionnaire (SDQ), completed by the parent or carer when the child was in Primary 1 (P1), the first formal year of schooling in Scotland when children are approximately five years old. We used validated cut-points (Goodman et al., 2000), designed to identify ‘normal’ (hereafter referred to as close to ‘average’) and ‘borderline/abnormal’ (hereafter referred to as ‘raised’) scores (Youth in Mind, 2016).

The childcare (mediator) measure was created using questions about childcare type used at 10 months, two, three and four years. Where children attended multiple childcare providers, the provider where the child spent the most amount of time was used. 23 different childcare types were reported but were grouped into seven core categories (see Appendix 1): Private group childcare; Local Authority or Community group childcare; Single person professional care i.e. nanny or childminder; Family or friends informal childcare; Grandparent informal childcare; Any other; None i.e. parental care only. It is these seven categories, at the four different ages, that were used in the sequence analysis to identify the most common longitudinal patterns of childcare use (see analysis section).

### 2.3 Covariate measures

Directed Acyclic Graphs (DAGs) were used to show the relationship between the variables of interest, and to identify any confounding that need to be considered in the analysis (Tennant et al., 2020). Two DAGs were created, one to show the relationship between childcare and mental health and the second to show the childcare as a mediator between SECs and mental health. VanderWeele (2019) ‘modified disjunctive cause criterion’ was used for confounder selection. Variables that are the cause of the exposure and outcome (referred to here as a confounder) or just the outcome (and are not caused by the exposure, referred to here as a covariate), are adjusted.

Covariates: Variables which were a cause of the outcome but which did not lie on the causal pathway were: child's sex (male/female) was considered a covariate in all analyses as it was only related to the outcome (mental health) and school adjustment was considered a possible covariate for children's mental health at the start of primary school. A school adjustment score was created using parental assessment of whether the child complained, was upset or reluctant, said good things and looked forward to school (low/high) (analyses confirmed that school adjustment was not affected by childcare).

Baseline confounders: variables that were thought to affect SECs and childcare and/or mental health included child's ethnicity (white/other ethnic background) and mother's age at 1st live birth (<20/20–29/30+).

Intermediate confounders: variables which could potentially impact on childcare and mental health included maternal attachment which was measured as a continuous score at baseline (when the child was approximately 10 months old), from six questions taken from the larger ‘Maternal Postnatal Attachment Scale’ (Condon & Corkindale, 1998) and two other questions assessing if the respondent had felt down or low since the birth of the child and how the respondent would describe themselves as a parent (range 8–48). *Time-varying* intermediate confounders included Maternal Mental Health, which was measured at baseline and five years, using the mental health component of the SF-12 Health Status Survey (continuous measure, ranging from 0 to 100). Composite, longitudinal measures were created for several variables which were measured across multiple sweeps. Child's general health, based on parental reports of very good/good/fair/bad/very bad general health from baseline to age four years (dichotomised to always good or very good vs. ever fair, bad or very bad), maternal employment measured from age 2 years until 5 years (always worked full-time/always worked part-time/always worked combination of full and part-time/combination of working and not working/never worked), and family composition spanning infancy to age five (always couple/always lone/mixture), Significant life events (such as divorce, bereavement and serious illness) up to age five were classified as significant life event(s)/no significant life events.

**Fig. 1:**
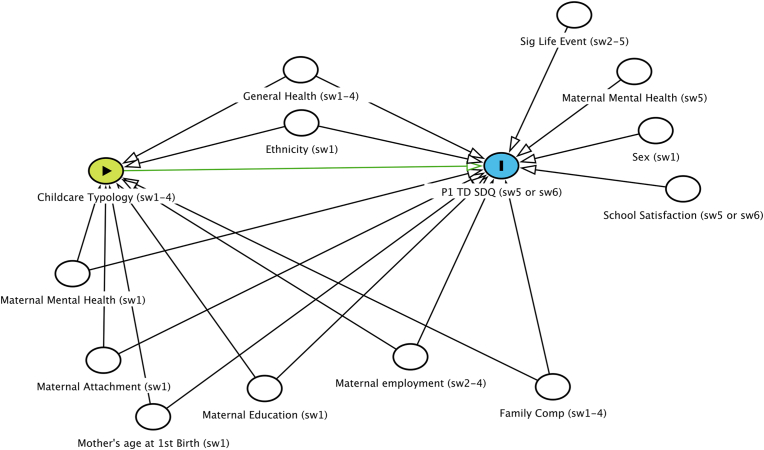
Directed Acyclic Graph showing the hypothesised relationship between childcare and mental health and potential confounding factors.

**Fig. 2:**
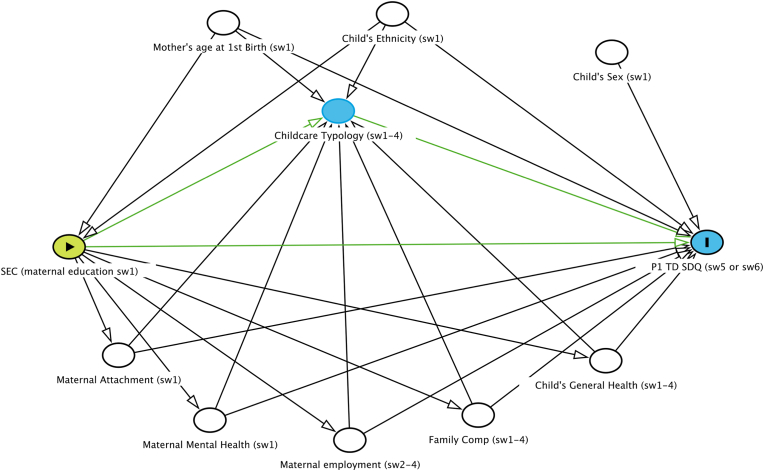
Directed Acyclic Graph showing childcare as a mediator between socio-economic circumstances and child mental health and potential confounders and covariates.

Because these intermediate variables were measured over the same or similar period as childcare, they may lie on the causal pathway between childcare and mental health (as opposed to being confounding the childcare-mental health relationship), and thus lead to overadjustment. Therefore, sensitivity analyses were run for the main mediation analysis, where only baseline (sweep 1) measures for each of these factors were adjusted for, referred to as the ‘partially adjusted’ analysis. DAGs created in Dagitty (Textor et al., 2017) that were used in the analysis can be found in Fig. 1, Fig. 2.

### 2.4 Sample design

**Fig. 3:**
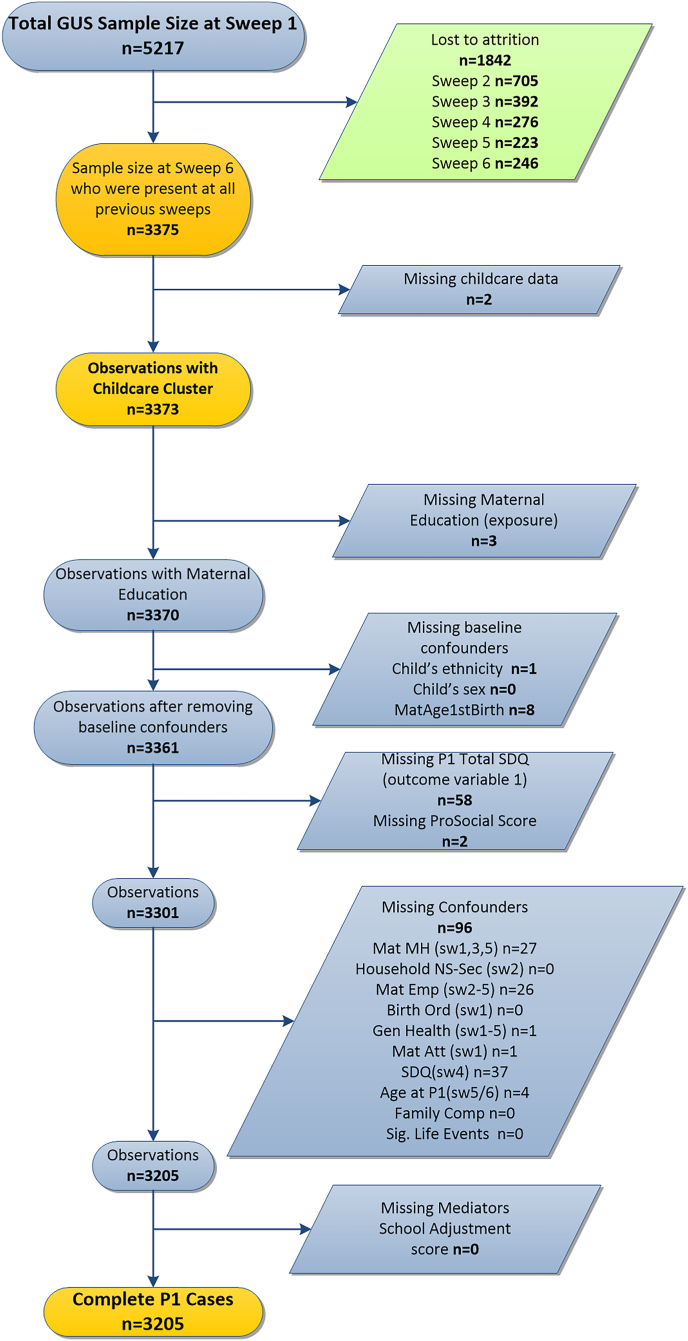
STROBE diagram to show the identification of the final analytic sample.

A complete case sample at the start of primary school was created n = 3205 (see Fig. 3), representing 61% of the original baseline sample respectively. Descriptive statistics for the samples compared with baseline can be found in Table 1. There were few differences in the characteristics of the two samples.

**Table 1:** Characteristics of the observed and analytic samples.

	Observed sample n (%) or mean (SE)	Analytic Sample n (%) or mean (SE)
BASELINE VARIABLES	n = 5217	n = 3205
**Maternal Education Level**		
Degree	1354 (26.0%)	846 (26.6%)
Highers	1556 (29.9%)	1015 (31.9%)
Upper Level Standard Grades	1372 (26.4%)	826 (26.0%)
Lower Level Standard Grades or No Quals	920 (17.7%)	491 (15.4%)
**Child's Ethnicity**		
White British	4965 (95.2%)	3043 (95.7%)
Other Ethnic Background	248 (4.8%)	136 (4.3%)
**Child's Sex**		
Male	2689 (51.5%)	1633 (51.4%)
Female	2528 (48.5%)	1546 (48.6%)
**Age of mother at 1st child's birth**		
Under 20	955 (18.3%)	547 (17.2%)
20–29	2675 (51.4%)	1621 (51.0%)
30 or older	1577 (30.3%)	1011 (31.8%)
**Family Type**		
Lone Parent	1059 (20.3%)	599 (18.8%)
Couple Family	4158 (79.7%)	2580 (81.2%)
**General Health**		
Very Good	3897 (74.7%)	2426 (76.3%)
Good	1014 (19.4%)	583 (18.3%)
Fair, Bad or Very Bad	305 (5.9%)	170 (5.3%)
**Childcare Use at 10 months**		
Private group childcare	649 (12.4%)	426 (13.4%)
LA or Community group childcare	110 (2.1%)	50 (1.6%)
Single person professional care	319 (6.1%)	212 (6.7%)
Family or friends informal childcare	302 (5.8%)	179 (5.6%)
Grandparent informal childcare	1699 (32.6%)	1090 (34.3%)
Any other	26 (0.5%)	16 (0.5%)
None	2111 (40.5%)	1210 (38.1%)
**Maternal Attachment Score**	39.63 (0.06)	39.53 (0.08)
**Maternal Mental Health**	50.00 (0.13)	50.06 (0.16)
SWEEP 2 VARIABLES	n = 4512	
**Maternal Employment Status**		
Not Working	1787 (39.7%)	1211 (38.1%)
Working Full Time	660 (14.7%)	475 (14.9%)
Working Part Time	2050 (45.6%)	1493 (47.0%)
PRIMARY 1 VARIABLES		
**Total SDQ Score at P1**		
Close to Average		2831 (89.1%)
Raised		348 (10.9%)

Table shows numbers and percentages or mean and standard error (SE) adjusted using sample weights therefore weighted n will differ from sample n.

### 2.5 Analysis

Using TraMineR (Gabadinho et al., 2011), childcare sequences were created for each child from age 10 months to around four years olds, consisting of the main childcare provision over the first four GUS sweeps. Children with similar sequences were grouped together using a method of cluster analysis called hierarchical clustering. This seeks to identify typologies which maximise similarities within typologies while ensuring there are meaningful differences between sequence typologies (Studer, 2013). A sequence tree in Appendix 2 shows how four clusters were identified (using ‘WeightedCluster’ in R (Studer, 2013)). These were assessed for stability using a bootstrap method used to validate the cluster solution (Hennig, 2007) and were considered to include a manageable number of groups, with sufficient sample sizes.

Multinomial regression was run to establish if there were differences between childcare typologies according to maternal education. “Survey” package (Lumley, 2020) in R was used to account for the complex survey design, that returns ‘model-robust’ standard errors, using Horvitz-Thompson type standard errors. These are a generalisation of the model robust ‘sandwich’ estimators. “svyVGAM” and in particular ‘svy_vglm’ function (Lumley, 2021) was used to run the multinomial regression producing odds ratios. Correct standard errors for relative risk (RR) regression models are achieved using the ‘*quasi-Poisson*’ function.

Marginal structural models were used to estimate the average treatment effect (ATE) of maternal educational qualifications on mental health, using logistic regression models to estimate odds ratios (ORs) and 95% confidence intervals (CI). We used inverse probability of treatment weighting (IPTW) to adjust for baseline confounders. These estimates represent observed levels of inequalities. Following this we estimated the controlled direct effect (CDE) where the mediator (childcare) is set to a fixed level for all respondents. More specifically, we examined two hypothetical scenarios where everyone in the population receives 1) Private group childcare, and 2) Single professional care. These scenarios were chosen because they represent commonly used childcare types at 3–4 years, which might be feasibly and universally extended to under 3s. For CDEs the inverse probably of treatment weights included a weight for the mediator, to adjust for intermediate confounders (see Appendix 3). An interaction between maternal education and childcare was included, to allow for the fact that the impacts of childcare might vary in different groups.

## 3 Results

### 3.1 Prevalence and inequalities in longitudinal patterns of childcare

At 10 months (sweep 1) 59.8% of all GUS families were using some form of childcare, this increased to 69.1% at around two years (sweep 2) and was 77.2% at about three years (sweep 3). By age four (sweep 4), 92.4% had taken up their universal childcare place, although this was not always the main childcare provider as 65.3% of families were using another form of childcare in addition to their universal childcare place (data not shown).

**Fig. 4:**
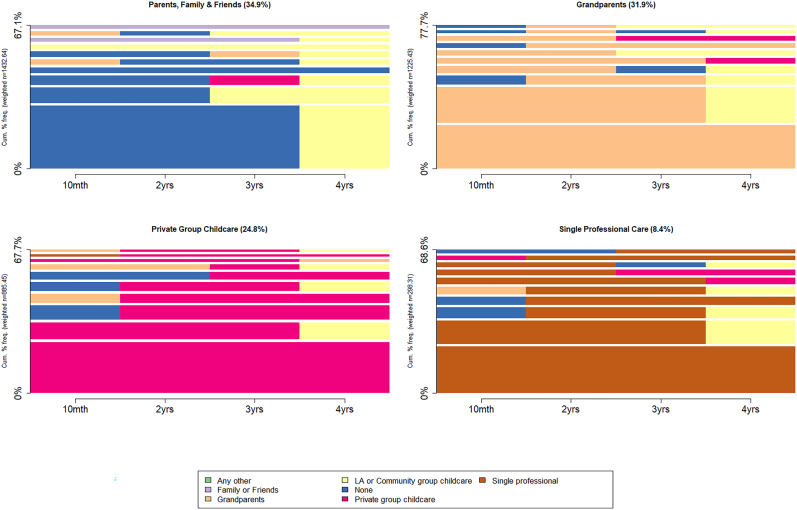
Most frequent sequences in the four childcare typologies.

Four typologies of the *main* childcare use throughout the early years were identified in the sequence analysis. These were labelled as: ‘Parents, friends & family’, ‘Grandparents’, ‘Private Group Childcare’, ‘Single Professional Care’. The most frequent combinations of individual sequences of childcare use, within each of the childcare groups, are represented in Fig. 4, with the different childcare types represented by different colours. Most children were using their universal childcare entitlement between three and four years either in local authority nurseries (e.g. attached to schools) in yellow or private nurseries in pink. Most of the variation in childcare usage occurred before the age three years.

**Parents, friends & family**’ (n = 1340, 34.9%) – in this group a large proportion of children are looked after at home (shown in blue), moving to a universal childcare place in local authority (yellow) when they are eligible, typically by age three or four years.

‘**Grandparents**’ (n = 1225, 31.9%) – in this group grandparents provide the bulk of the care (shown in orange), with most children moving to a universal childcare place when they were eligible. However, a sizeable proportion remained classified as grandparent care, indicating that grandparents were still providing the majority of childcare on a weekly basis even when the universal place is available.

‘**Private Group Childcare**’ (n = 953, 24.8%) – in this group, private providers (e.g. private nurseries, preschools, shown in pink) are the main childcare type. Some switch to local authority provision (yellow) when they become eligible for their universal childcare place although many remained with private providers (which could also offer the free entitlement).

‘**Single Professional Care**’ (n = 324, 8.4%) – in this smallest group, children are initially looked after by a childminder or nanny (shown in brown). Again, children typically move to a universal childcare place provided by a local authority (yellow) when they are eligible, but some continue to receive their universal childcare from a single professional. The prevalence of the different childcare types remained similar in the complete case sample (Appendix 4).

Compared to parent, family and friends care, the odds of children having private group childcare were lower among those whose mothers had lower educational qualifications, with the lowest risk in those with whose mothers had lower level Standard Grades or no qualifications (OR 0.12 (95% CI: 0.09–0.18). A similar pattern was seen for single professional care, with the lowest risk again for those children whose mothers had lower level Standard Grades or no qualifications (OR 0.09 (95% CI: 0.05–0.17)) (Table 2). While there is some evidence of lower odds of grandparent care by maternal education, these are smaller by comparison to the other childcare types. Children in lower socioeconomic circumstances appear to have mostly informal or no childcare in place before accessing universal childcare.

**Table 2:** Inequalities in longitudinal typologies of childcare.

	Childcare Typology		
	Odds Ratios (95% CIs)		
	Grandparent^a^	Private Group^a^	Single Professional^a^
**Maternal Education Level**			
Degree (ref)	–	–	–
Highers	0.914 (0.725–1.152)	0.457 (0.366–0.571)	0.443 (0.320–0.614)
	p = 0.445	p < 0.001	p < 0.001
Upper Level Standard Grades	0.625 (0.494–0.789)	0.211 (0.157–0.284)	0.267 (0.168–0.424)
	p < 0.001	p < 0.001	p < 0.001
Lower Level Standard Grades or none	0.368 (0.271–0.501)	0.123 (0.085–0.178)	0.089 (0.045–0.173)
	p < 0.001	p < 0.001	p < 0.001
Observations	3205	3205	3205

OR - Odds Ratios calculated using multinomial regression with 95% Confidence Interval.

a Adjusted for Ethnicity.

### 3.2 The relationship between childcare and mental health

Appendix 5 shows the proportions of children with raised TD scores according to childcare type. In the fully adjusted analysis (Table 3), private group childcare was associated with a lower risk of having a raised TD score (RR: 0.83 (95% CI: 0.62–1.12)), although with relatively wide confidence intervals. The partially adjusted model (which accounted only for baseline confounders, in case of overadjustment) showed similar results, Table 3.

**Table 3:** Risk of raised Total Difficulties Score according to longitudinal typologies of Childcare.

	Raised P1 Total Difficulties Score		
	Risk Ratios (95% CIs)		
	Unadjusted	Partially Adjusted^a^	Fully Adjusted^b^
**Childcare Typologies**			
Parent, Family & Friend Care (ref)	–	–	–
Grandparent Care	0.754 (0.564–1.009)	0.973 (0.716–1.323)	1.052 (0.775–1.429)
	p = 0.063	p = 0.865	p = 0.746
Private Group Childcare	0.551 (0.404–0.752)	0.786 (0.567–1.090)	0.832 (0.618–1.119)
	p < 0.001	p = 0.157	p = 0.231
Single Professional Care	0.534 (0.351–0.813)	0.785 (0.515–1.197)	0.770 (0.505–0.173)
	p = 0.005	p = 0.267	p = 0.231
Observations	3205	3205	3205

RR - Risk Ratios calculated using logistic regression with quasipoisson log function with 95% Confidence Interval.

Both adjusted models are adjusted for sex, ethnicity, maternal education level, maternal attachment, family composition (sw1-4), mother's age at 1st birth and school satisfaction at P1 transition.

Sw = sweep.

a Time-fixed confounders – maternal mental health (sw1), general health (sw1), maternal employment (sw2).

b Time-varying confounders – maternal mental health (sw1 & sw5), general health (sw1-4), maternal employment (sw2-4) and significant life events (sw2-5).

Single professional care was associated with a lower risk of having raised TD, with a fully adjusted RR of 0.77 (95% CI: 0.51–1.17). This typology had the widest 95% confidence intervals but this is likely to be driven by the smaller number of observations in this group (n = 257, 8.1% of weighted analytic sample). The partially adjusted model which excluded time-fixed confounders, in case of overadjustment, was not meaningfully different from the fully-adjusted models. Grandparent care was not associated with a risk of raised TD compared to parental care, with a RR very close to 1.

### 3.3 Estimating the impacts of hypothetical childcare scenarios on inequalities in mental health

The first column of Table 4 shows a social gradient in children's mental health (estimated by an Average Treatment effect, ATE). Children whose mothers have upper level Standard Grades are 2.6 (OR, 95% CI: 1.63, 4.14) times as likely to have a raised TD score with children of mothers who have low level Standard Grades or no qualifications more than three times as likely (3.18 (95% CI: 1.88, 5.37)).

**Table 4:** Inequalities in Mental Health at P1 and Before and After setting Childcare at certain Levels (representing hypothetical scenarios).

	Raised P1 Total Difficulties Score		
	ATE^a^	Odds Ratios (95% CI) CDE^b^ Private Group Childcare	CDE^c^ Single Professional Care
**Maternal Education Level**			
Degree or above	–	–	–
Highers	1.352 (0.812, 2.251)	2.164 (0.951, 4.927)	0.713 (0.122, 4.156)
	p = 0.251	p = 0.072	p = 0.709
Upper Level Standard Grades	2.596 (1.630, 4.136)	3.600 (1.399, 9.264)	1.782 (0.308, 10.293)
	p < 0.001	p = 0.011	p = 0.522
Low Level Standard Grades or none	3.181 (1.883, 5.374)	3.778 (1.463, 9.759)	2.420 (0.204, 28.759)
	p < 0.001	p = 0.009	p = 0.488
Observations	3205	3205	3205

OR - Odds Ratio calculated using quasibinomial log function with 95% Confidence Interval.

The CDE and ATE estimates assume no residual confounding or reverse causation.

a ATE: Average Treatment Effect, i.e., the estimated average effect of each exposure, after adjustment for pre-exposure confounders.

b CDE: Controlled Direct Effect, i.e., an estimate of the effect of each exposure when the mediator is held constant at Private Group Childcare.

c CDE: Controlled Direct Effect, i.e., an estimate of the effect of each exposure when the mediator is held constant at Single Professional Care.

In the hypothetical scenario where all children receive private group childcare (second column of Table 4), inequalities in mental health generally increased. For example, the odds ratio for raised TD scores among children whose mothers have low level Standard Grades or no qualifications increased from 3.18 (95% CI: 1.88–5.37) reported previously, to 3.78 (95% CI: 1.46–9.76). Inequalities also widened for the intermediate groups of maternal educational qualifications.

In a second hypothetical scenario, where all children were looked after in single professional care (third column of Table 4), there was a decrease in inequalities. For example, among children whose mothers have low level Standard Grades or no qualifications, the OR fell to 2.24 (0.2, 28.8), although the confidence interval is wide. This indicates that, while inequalities still exist, in a counterfactual situation in which all children were to receive the single professional care, inequalities in child mental health could potentially be considerably reduced. However, the very wide confidence intervals would suggest that there is still a lot of uncertainty and these results require replication elsewhere.

## 4 Discussion

### 4.1 Summary of findings and comparisons with other research

Four main longitudinal patterns, or typologies, of childcare throughout the early years were identified; these mainly differentiated between childcare types used before the commencement of universal childcare provision in Scotland (which almost all children used). Children in the lowest socioeconomic circumstances, assessed by maternal education, were less likely to use private group childcare or care from single professionals than children whose mothers had degrees. While the longitudinal patterns of childcare use identified were created in one specific cohort (GUS), the findings are in line with existing UK and international research on inequalities in childcare use. For example, in other European countries, children below the age of three years from the lowest socioeconomic circumstances are also far less likely to have formal childcare than children in the medium and highest socioeconomic circumstances (Adema et al., 2016). We found that when exposed to grandparent care before the start of universal childcare uptake, children's likelihood of poor mental health at the start of primary school did not differ substantially from care by parents, family & friends. Melhuish and Gardiner (2021) found that children with high levels of informal childcare usage (that included grandparent care) between the ages of two and four years had slightly higher SDQ TD scores than children cared for at home with parents. These differences in findings may be related to the intensity of childcare - Melhuish and Gardiner observed this negative impact among children who were looked after in informal childcare for over 20 h per week. In GUS, the majority of children in the grandparent childcare group transitioned into centre-based childcare at age three, and prior to this age the average time spent in main childcare type was far below 20 h (median hours ranged from five to 9 h per week). Another explanation for the difference may be Melhuish and Gardiner's inclusion of other carers in the informal childcare group (which included friends, other family members and nannies, although grandparents were the predominant group).

Given that childcare is intended to create more equal outcomes, we examined the hypothetical impact of different childcare scenarios on inequalities in children's mental health. The results suggested that if everyone was given private group childcare before the universal entitlement at age three to four years in Scotland, inequalities in mental health may increase. This concurs with some of the evidence that centre-based childcare before the age of three years can have a detrimental effect on mental health (Berger et al., 2021; Loeb et al., 2007; Yamauchi & Leigh, 2011), particularly if the quality of childcare is low (Melhuish et al., 2015). Previous research has suggested that poor quality childcare can be detrimental to socioemotional outcomes for children in lower socioeconomic circumstances (Melhuish et al., 2015; Pluess & Belsky, 2009). However other research suggests that inequalities in externalising and internalising symptoms, by parental education, could be attenuated in the UK in a scenario where there was universal use of centre-based care before the age of three (specifically between 26 and 31 months) (Green et al., 2021). Outcomes were measured when children were three years old and it is unclear whether any effects or any impacts on inequalities would persist at age 5 (as considered in our study) or whether differences in the timing of exposure are important. It is also possible that the differences in these findings are driven by different contexts in Scotland as compared to the whole of the UK.

While we found that universal uptake of group childcare might widen inequalities, we found that universal uptake of single professional care (e.g. childminders or nannies) might lead to a small reduction in inequalities in mental health, although there was a lot of uncertainty around this scenario since single professional care was relatively rare in the GUS children. There has been very little research into childminders and other home-based childcare (Ang et al., 2017; Dunlop, 2017) therefore direct comparison with other research is not possible. Childcare might contribute to health inequalities via *differential exposure* (i.e. if childcare types which are more supportive of health are more prevalent in advantaged groups). We found this to be the case in the current analysis, with children from less advantaged backgrounds less likely to be exposed to private or single professional care. Additionally, childcare may also widen or narrow inequalities via *differential susceptibility* (for example if the benefits of childcare were greater for more compared to less advantaged groups), which may occur, for example, if quality varies between different socio-economic groups. One study found that infants with difficult temperaments experienced more behaviour problems later on in childhood if they were exposed to low quality childcare (Pluess & Belsky, 2009). The analysis presented here allowed for this possibility of differential susceptibility, by including an interaction between SECs and childcare, but could not quantify the contribution of differential susceptibility specifically, or investigate the role of childcare quality. It is plausible that in the Scottish context presented here, the potential widening of inequality we found in the scenario of universal group-based childcare might be explained by the fact that children from less advantaged backgrounds are exposed to poorer quality group childcare.

It has been suggested that nurturing environments have been found to enhance the outcomes of vulnerable children to a greater extent than those children who were not considered vulnerable (Belsky et al., 2007). This could explain our finding that universal uptake of single professional care, prior to universal childcare at the age of three years, could decrease inequalities in mental health between the most and least deprived. In their review of research on childminding, Ang et al. (2017) recognise that home-based childcare is distinct from other types of provision. Because it is provided in homes, there is more heterogeneity in the physical setting and also the activities provided vary by provider. Therefore, while there is some limited evidence that for very young children in Scotland this type of childcare has the potential to reduce inequalities, the heterogeneous nature of the provision may also have the potential to widen inequalities if attention was not paid to the type and quality of that single professional care. This would benefit from replication in other studies.

The evidence presented is based on mental health outcomes to the exclusion of other outcomes which are known to benefit from early years education and childcare, including cognitive development. Therefore, when contemplating the expansion of universal childcare provision, there will need to be a balance between the benefits to cognitive and developmental skills and the impact on socioemotional outcomes for children as well as other aspects of health which may be affected by childcare, such as overweight and obesity (Black et al., 2017; Pearce et al., 2010). We found that certain types of childcare have the potential to reduce, but not eliminate, inequalities in mental health. Meaning that what is left is the effect of socioeconomic inequality through already established mechanisms such as poor attachment, low emotional capacity, and ultimately the lack of financial resources and familial adversity (Pearce et al., 2019).

### 4.2 Strengths & weaknesses

One of the strengths of this research is that it captures main childcare over the first four years of life which, to our knowledge, has not been done in any other studies. The use of sequence analysis in creating childcare typologies, incorporating both formal and informal childcare over a period of four years, is novel and was possible only due to the frequency of early years sweeps in the GUS data. However, only the most dominant childcare type at each timepoint was measured with other concurrent childcare types being ignored. This was because the number of possible permutations of different childcare variations would have made sequence analysis, and interpretability, difficult. While the typologies were assessed to be stable and have enough heterogeneity between the four groups, some nuances of some childcare combinations used by families will have been lost.

Like many longitudinal cohort studies, GUS is subject to attrition, although the characteristics of analytical sample did not differ substantially from the GUS cohort after the sample and response weights were applied. Another limitation was the small size of the single professional childcare typology which accounted for 8.1% (n = 274) of the weighted analytic sample. This has led to relatively large uncertainty around some estimates and the need for these questions to be addressed in larger datasets.

While every effort was made to account for variables that could have been confounders, residual confounding cannot be ruled out. For example, we could not adjust early child mental health or temperament which may have influenced childcare type. There may also be measurement error in the confounders that we did adjust for.

The causal ordering for some variables may be unclear. For example, we have assumed that maternal employment, which was measured across sweeps 2–4, affected childcare choice (making it an intermediate confounder of childcare and TD) as opposed to the reverse (it lying on the causal pathway between childcare and TD). If these intermediate confounders were instead mediators, then we would expect that the true estimate would lie somewhere between the partially adjusted and fully adjusted models. We therefore carried out a sensitivity analysis which excluded adjustment for intermediate confounders. As noted in the results, these results did not differ meaningfully from the fully adjusted model. GUS did not contain information on the quality of childcare, which would provide crucial context to these findings and potentially explain the results of the hypothetical scenarios.

The early years’ data on childcare were collected between 2005 and 2009 in GUS, when universal childcare provision for three-to four-year-olds was 12.5 h per week. Since then, universal provision has increased to 16 h per week (in 2014) and to 30 h per week (in 2021) in Scotland. Thus, our findings may not be the same for current or future cohorts of children who experience greater doses of universal childcare provision in the early years. Additionally, the quality of universal childcare may have improved over the intervening period via the implementation of the Curriculum for Excellence in Scotland in 2010 (Scottish Government, 2022a). However, the national curriculum applies to universal childcare provision for three to four year olds, and so the findings presented here are still important for highlighting potential policy considerations for extending universal childcare entitlement to younger ages.

### 4.3 Implications for policy and future research

In 2021, the Scottish Government (2021) in their strategy plan ‘A Fairer, Greener Scotland: Programme for Scottish Government, 2021–22’ stated that they will seek to engage with families, early learning providers and academics to understand how best to expand the funding of ELC to one and two year old children in low income households in Scotland. The UK Government have also recently announced their intention to expand the 30 h of universal childcare for working parents in England to children aged nine months to two years (HM Treasury, 2023). The findings here suggest that the scaling up of private group childcare before the age of three years, if experienced in the same was as by the GUS children, has the potential to widen mental health inequalities. Single professional care, for example from childminders, in the very early years, may have the potential to decrease the social gradient in poor mental health and wellbeing, although these findings require replication elsewhere due to small sample sizes. Careful consideration will need to be given to the type and quality of childcare provision available to one and two years olds, especially for children experiencing less advantaged socioeconomic circumstances. Future research should take a more nuanced approach and consider which aspects of childcare quality are beneficial to mental health across different socio-economic groups. Quality ratings for single professional care, e.g. for registered childminders, may give an insight into how structural or process aspects of childcare quality may impact on mental health outcomes. There will also need to be consideration given to balance the benefits for other outcomes, such as cognitive development, for which there is currently a more solid evidence base.

In conclusion, universal provision of early learning and childcare has been implemented in many high income countries as a means to improve life chances and reduce inequalities. The analysis presented here used a novel approach to capture children's early childcare experiences in Scotland and to estimate the potential impacts of hypothetical childcare policy scenarios on mental health inequalities. Findings indicate that care by single professionals could attenuate some of the risks of poor mental health associated with low socioeconomic circumstances in the early years; while group-based childcare, as experienced by a particular cohort of children born in Scotland 2004–5, could increase these risks if universally rolled-out to children under three years of age. The quality of childcare providers is likely to play an important role and should be considered closely when rolling out any childcare policy. This is especially important when considering childcare provision for children in the lowest socioeconomic circumstances.

## Funding

ER and AP are supported by funds from the 10.13039/100010269Wellcome Trust [205412/Z/16/Z] and a University of Glasgow Lord Kelvin/Adam Smith leadership fellowship. ER, AL, and AP are supported by funds from the 10.13039/501100000265Medical Research Council [MC_UU_00022/2] and the Scottish Government Chief Scientist Office [SPHSU17].

## Funding

This work was supported by the 10.13039/501100000265Medical Research Council (MC_UU_00022/2), the Chief Scientist Office (CSO) (SPHSU17), the 10.13039/100010269Wellcome Trust (205412/Z/16/Z) and a University of Glasgow Lord Kelvin/Adam Smith leadership fellowship.

## Ethical statement

Ethical approval was received from a Research Ethics Committee at each study sweep. The present secondary data analyses did not require additional ethics approval.

## CRediT authorship contribution statement

**Elaine Robertson:** Conceptualization, Data curation, Formal analysis, Methodology, Visualization, Writing – original draft, Writing – review & editing. **Alastair Leyland:** Methodology, Supervision, Writing – review & editing. **Anna Pearce:** Conceptualization, Methodology, Supervision, Writing – original draft, Writing – review & editing.

## Figures and Tables

**Table:** 

Group		Childcare	Label
1		Private creche or nursery	Private group childcare
		Private playgroup	
		Workplace creche	
		Nursery class attached to an independent school	
2		LA Playgroup or Preschool	LA or Community group childcare
		LA Creche or nursery	
		Community or voluntary playgroup/nursery	
		Nursery class attached to primary school	
		Family centre	
3		Daily visiting nanny	Single person professional childcare
		Live-in nanny	
		Childminder	
		Child carer (provided via childcare agency)	
4		Ex-spouse or partner	Family or friends informal childcare
		Another Relative	
		Friend or neighbour	
		Babysitter who comes to house	
		Child's older sibling	
5		Grandparents	Grandparent informal childcare
6		Any other childcare provider	Any other
7		None	None

**Figure:**
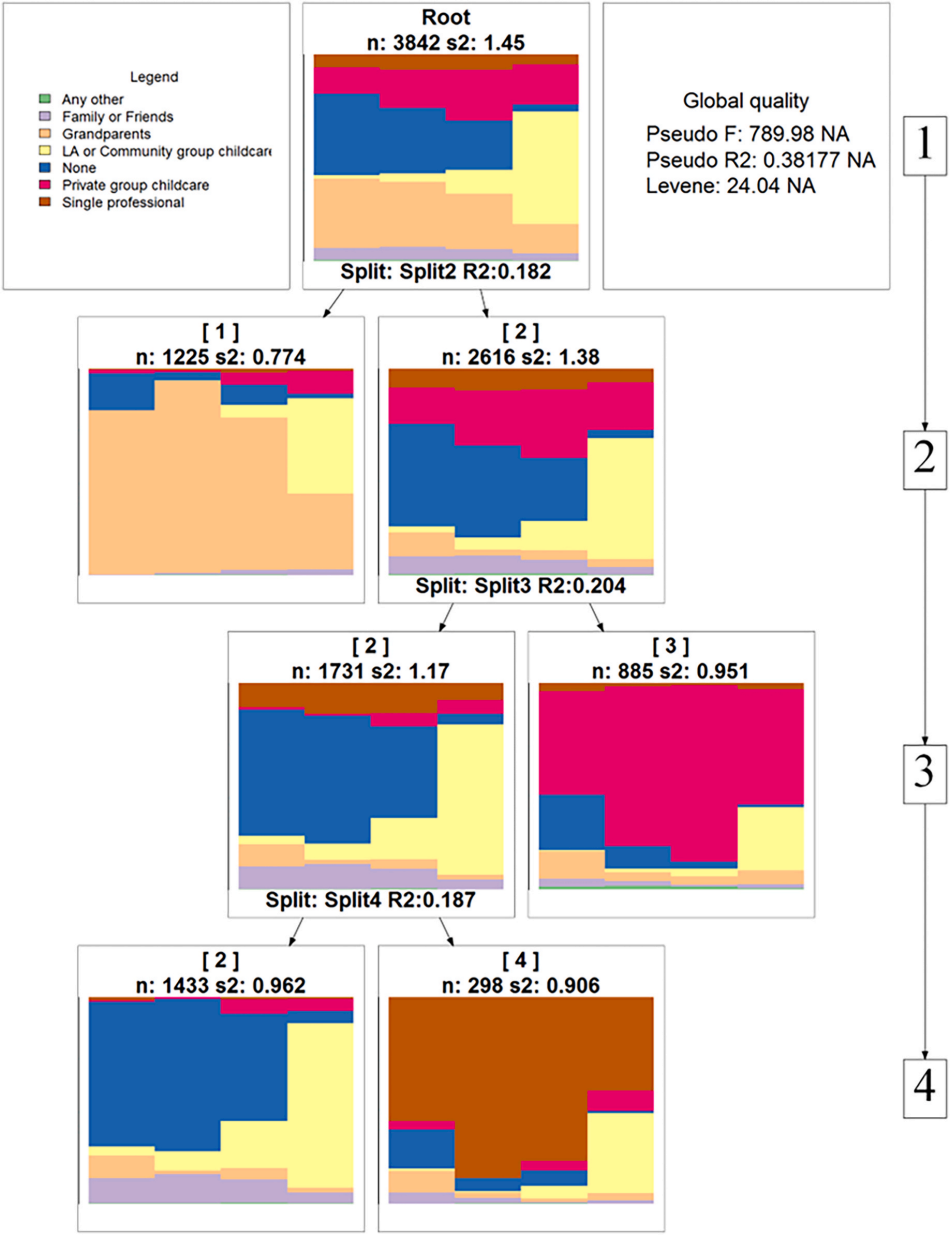


**Figure:**
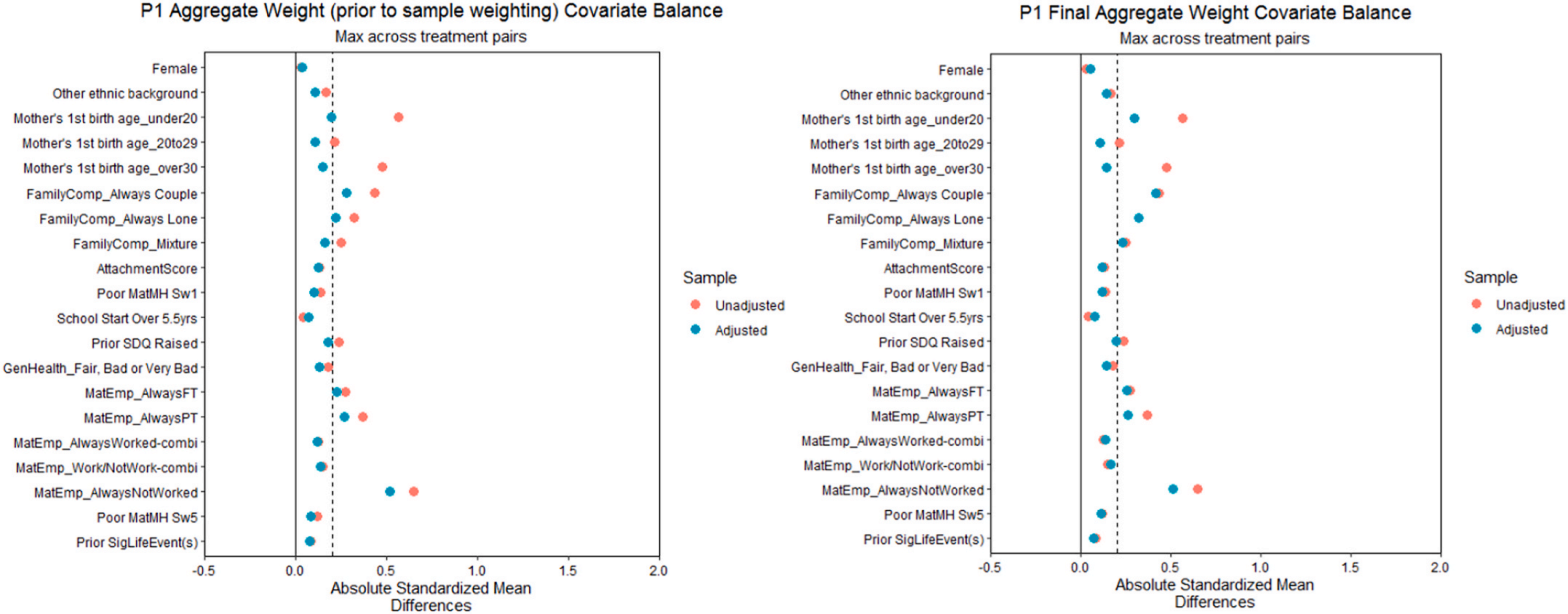


**Table:** 

	Initial Sample* Observations n (%)	Initial Sample* Observations weighted n (%)	P1 Sample n (%)	P1 Sample weighted n (%)
DERIVED CHILDCARE VARIABLE	n = 3842	n = 3841	n = 3205	n = 3179
**Childcare Typology**				
Parents, Family & Friends	1340 (34.9%)	1433 (37.3%)	1068 (33.3%)	1138 (35.8%)
Grandparents	1225 (31.9%)	1225 (31.9%)	1045 (32.6%)	1038 (32.7%)
Private Group Childcare	953 (24.8%)	885 (23.0%)	810 (25.3%)	746 (23.5%)
Single Professional Care	324 (8.4%)	298 (7.8%)	282 (8.8%)	257 (8.1%)

*Initial sample includes those children who were present in sweeps 1–4 and who had no missing childcare data.

**Table:** 

	P1 SDQ TD	
	Close to Average	Raised
Parents, Family & Friends	978 (85.9%)	160 (14.1%)
Grandparent Care	927 (89.4%)	110 (10.6%)
Private Group Care	688 (92.2%)	58 (7.8%)
Single Professional Care	238 (92.6%)	19 (7.4%)

SDQ: strengths and difficulties.

**Table:** 

	Mean SDQ TD (weighted sample)	Median SDQ TD (weighted sample)	IQR (weighted sample)
Parents, Family & Friends	7.9	6.5	7
Grandparent Care	7.4	6.5	6
Private Group Care	7.1	6.5	5
Single Professional Care	6.8	5.5	5

## Data Availability

The data that has been used is confidential.
